# The gain of hydrogen peroxide resistance benefits growth fitness in mycobacteria under stress

**DOI:** 10.1007/s13238-014-0024-5

**Published:** 2014-02-22

**Authors:** Xiaojing Li, Jun Tao, Jiao Han, Xinling Hu, Yuling Chen, Haiteng Deng, Guangchun Zhang, Xiaoxiang Hu, Kaixia Mi

**Affiliations:** 1 CAS Key Laboratory of Pathogenic Microbiology and Immunology, Institute of Microbiology, CAS, Beijing, 100101 China; 2 University of Chinese Academy of Sciences, Beijing, 100082 China; 3 School of Life Sciences, Tsinghua University, Beijing, 100084 China; 4 School of Life Sciences, China Agriculture University, Beijing, 100094 China; 5 Beijing Key Laboratory of Drug Resistance and Resistome, Beijing, 100101 China

**Dear Editor**,

Tuberculosis (TB) is a formidable challenge to global health and is caused by an ancient pathogen, *Mycobacterium tuberculosis* (WHO, 2010). In the pre-antibiotic era, TB killed 20% of adults in Europe and North America in the period from the seventeenth to the nineteenth centuries (Wilson, 2005). Since antibiotics have been included in TB treatment regimens, millions of human lives have been saved by this chemotherapy. However, the standard anti-tuberculosis therapy is only effective for drug-susceptible TB but not for multi-drug resistant and extensively drug-resistant TB. Unfortunately, our knowledge of drug resistance in mycobacteria remains unclear and thus hampers the development of new anti-tubercular drugs. The basic mechanisms of drug resistance in mycobacteria are complicated and the outcome of drug resistance stems from evolution in the final analysis. Understanding the evolutionary strategies used by *M. tuberculosis* might give new insights into designing control strategies against *M. tuberculosis* (Little et al,. 2012).

As the most successful bacterial pathogen, *M. tuberculosis* has evolved elegant strategies to evade a host’s immune defensive system. For example, D’Arcy Hart and colleagues observed that *M. tuberculosis*-containing phagosomes do not fuse with the lysosome inside murine macrophages (Armstrong and Hart, 1971). Several studies indicated that *M. tuberculosis* succeeds as an intracellular pathogen is partially due to its ability to survive and persist inside macrophages, which provide hostile environments with starvation and other stresses (Podinovskaia et al., 2013). When *M. tuberculosis* infects macrophages, the mycobacteria must overcome exogenous reactive oxygen species (ROS), one of the classical innate defense mechanisms against infection. Additionally, during latency, *M. tuberculosis* continues to be under oxidative stress; thus, the accumulation of mutations caused by oxidative DNA damage was predicted to be a potential risk for drug resistance (Ford et al., 2011). Recently, studies have shown a correlation of ROS damage of DNA with antibiotic resistance (Kohanski et al., 2010), although this notion has been disputed by other recent studies that argued that antibiotic exposure did not induce the formation of ROS (Keren et al., 2013). The role of ROS in the evolution of drug resistance is still an open question.

Currently, the view of evolution is mostly derived from genomic sequencing and lacks direct experimental evidence. In this study, to explore the correlation between hydrogen peroxide (H_2_O_2_) resistance and growth benefits in mycobacteria under stress, we developed a model for evolutionary screening of H_2_O_2_-resistant mutations in a pathogenic mycobacteria model, *M. smegmatis*, using a clinically important stressor, H_2_O_2_ (Fig. 1). This strategy of screening for H_2_O_2_-resistant mutations under increased H_2_O_2_ conditions in *M. smegmatis* was chosen to allow genome-wide identification of essential genes involved in oxidative stress because setting up a library of transposon mutants would fail, by default, to identify candidates that are essential genes. To demonstrate that resistance phenotypes resulted from chromosomal mutations, we continuously sub-cultured resistant strains for 10 generations without H_2_O_2_ treatment. The actual minimum inhibitory concentrations (MICs) of H_2_O_2_ in different mutants selected under different H_2_O_2_ concentrations were different, indicating that gradually increasing H_2_O_2_ resistance was due to the accumulation of mutated genes rather than a key mutated gene that caused the resistance to H_2_O_2_ (Fig. S1). After 54 days of selection, the MIC of the mutant strains with the maximum resistance to H_2_O_2_, selected at an H_2_O_2_ concentration of 1.5 mmol/L, was 3.125 mmol/L, while that of the wild type strain mc^2^155 under the same conditions (7H9 medium with 10% ADS) was 0.039 mmol/L. We named the mutant strain mc^2^51. As evolutionary cross-stress protection was observed under various stresses, we compared the growth advantage between mc^2^51 and mc^2^155 in corresponding stresses. We first measured the doubling time of both wild type mc^2^155 and mutant strain mc^2^51. A significant difference in doubling time was detected: the doubling time of wild type mc^2^155 was 2.734 ± 0.051 (*n* = 30), while the doubling time of mc^2^51 was 3.448 ± 0.087 (*n* = 30) (Fig. 2B), and the generation time increased up to 126%, compared to wild type mc^2^155. A growth advantage was observed for mutant strain mc^2^51 in M9 minimal medium compared to wild type mc^2^155, but this did not occur in M9 minimal medium with 10% ADS, suggesting that the mutant strain has a growth advantage under starvation conditions. Additionally, to investigate whether mc^2^51 has a survival advantage in host cells, cell line THP1 was infected with either mutant strain mc^2^51 or wild type strain mc^2^155 at an MOI of 1:10. The mutant strain mc^2^51 had a statistically significant growth advantage in macrophages compared to wild-type mc^2^155 (Fig. 2D). As a correlation of ROS damage and antibiotic resistance was indicated, we also detected the MICs of various antibiotics in both mc^2^155 and mc^2^51. Previous studies have suggested that H_2_O_2_ affected *KatG* mutation frequency and caused changes in resistance to isoniazid (INH) (Timmins and Deretic, 2006). A 100-fold difference in the MIC of INH was detected between mc^2^155 and mc^2^51 (Table S1). To provide insight into the mechanisms involved, we sequenced the whole genomes of both mutant strain mc^2^51 and wild type mc^2^155. Compared with mc^2^155, there are 22 single nucleotide polymorphisms (SNPs) in the mc^2^51 genome, of which 19 are located in coding regions, and 12 of these 19 SNPs are nonsynonymous mutations (Table S2). The data showed that G:C > T:A transversion and G:C > A:T transition were the major mutation types (Fig. 2E). The ratio of G:C > T:A transversion to G:C > A:T transition is an index to measure the contribution of oxidative DNA damage to the development of antibiotic resistance; it was 0.7 in mc^2^51 but is 0.25 under normal growth conditions. This increased ratio indicated that oxidative DNA damage caused the DNA mutations of mc^2^51. It should be noted that the ratio for mutations of *M. tuberculosis* isolated from macaques with latent infection increased drastically to 0.67 (Ford et al., 2011), indicating that oxidative DNA damage is a dominant source of mutation during both active and latent phases of *M. tuberculosis*. To further determine the possible roles of those genes in H_2_O_2_ resistance, the sequences of the corresponding genes amplified from wild type mc^2^155 genomic DNA were cloned into the integrating vector pMV361 (Table S4) and introduced into mc^2^51 via electroporation. Surprisingly, there are no changes in the MIC of H_2_O_2_ for most detected transformed strains, except for one strain of mc^2^51 with pMV361-*fur*(*msmeg_3460*) (Table S3 and data not shown). Furthermore, because *fur* is located upstream of *msmeg_3461*, which encodes KatG, the mutated Fur (Ferric uptake regulation protein) with substituted A28V would influence KatG expression. Increased peroxidase and catalase activities were found in mc^2^51 (Fig. 2F), and KatG was confirmed by mass spectrometry analysis (Fig. 2F).

**Figure 1:**
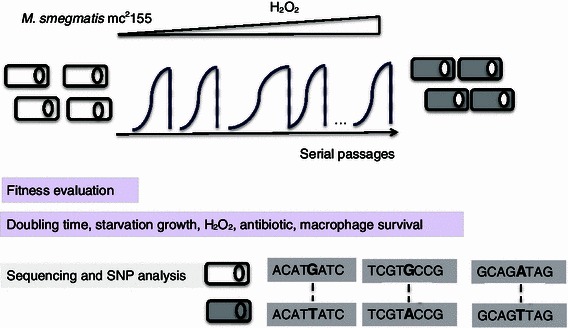
Overview of the experimental set-up. *M. smegmatis* mc^2^155 evolved for 25 generations under increasing H_2_O_2_ in 7H9 medium supplemented with 10% ADS (50 g/L albumin, 20 g/L dextrose and 8.1 g/L saline). The growth fitness of the final evolved strain was determined by doubling time, growth curve, starvation growth, survival under H_2_O_2_ stress, antibiotic resistance, and within macrophage-like cell line THP-1. The genomic DNA of both wild type mc^2^155 and mutant strains mc^2^51 was sequenced to identify single nucleotide polymorphisms (SNPs) in the mutant strain

**Figure 2:**
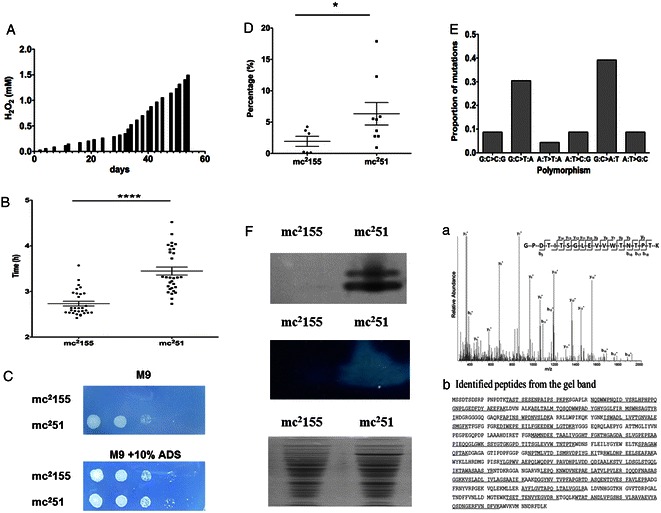
Characterization of H_2_O_2_ mutant strain mc^2^51. (A) The concentrations of H_2_O_2_ corresponding to the selected date throughout the whole selection process. (B) The H_2_O_2_-resistant strain mc^2^51 showed reduced growth compared to wild type mc^2^155. The growth rate is indicated by doubling time, which was measured using a fluctuation assay. (C) Growth advantage was determined between mc^2^155 and mc^2^51. The three panels represent serial dilutions (1:10) of mc^2^155 and mc^2^51 on M9 minimal medium or M9 minimal medium supplemented with 10% ADS, respectively; cultures were spotted (5 μL) onto the indicated medium. Photographs were taken after 3-day incubation. (D) The H_2_O_2_-resistant mutant strain mc^2^51 has a survival advantage over wild type mc^2^155 in the macrophage-like cell line THP-1. (E) The proportion of polymorphisms of nucleotide pair mutations from single nucleotide polymorphisms (SNPs). A total of 22 mutations were identified in mc^2^51 compared to wild type mc^2^155. G:C > T:A transversion and G:C > A:T transition are the major mutation types. (F) The activities of peroxidase and catalase in mc^2^51 and mc^2^155 and the identification of KatG using mass spectrometry. Whole lysates including approximately 50 μg protein were separated by non-denaturing 7.4% polyacrylamide gels. By searching the *M. smegmatis* protein database, an MS/MS spectrum matched GPDTITSGLEVVWTNTPTK. The highlighted peak is specific to the sequence of KatG. The underlined peptides were identified by MS/MS searching of the band covering 68% of the KatG sequence

As an intracellular pathogen, the success of *M. tuberculosis* is partially due to its ability to survive and persist in macrophages. For example, ROS produced by the host is one of the most important classical innate defense mechanisms against infection. To defend against ROS stress, mycobacteria have evolved many detoxification strategies to scavenge H_2_O_2_ (Kumar et al., 2011). Several studies have suggested that accumulation of mutations might link to the emerging drug resistance. In this study, we first selected an H_2_O_2_-resistant strain, mc^2^51, which displayed an 80-fold higher MIC of H_2_O_2_ than that in wild type mc^2^155. The mutant strain mc^2^51 has a growth advantage under starvation conditions and within host macrophage cells, but no resistance or susceptibility to the many investigated antibiotics except INH. To characterize the correlation of SNPs in mc^2^51 with the adaptive evolution of *M. smegmatis* resistance to H_2_O_2_, we sequenced the genome of mc^2^51 and showed that the SNP in *fur* contributed the observed resistance to H_2_O_2_ (Table S3). Furthermore, the mass spectrometry results indicated that the mutation of *fur* changed the expression of *katG*, causing high resistance to H_2_O_2_. Our results showed that the development of H_2_O_2_ resistance in mycobacteria led to the gain of growth benefits in starvation conditions and within macrophages.

The ratio of G:C > T:A transversion to G:C > A:T transition mutation is used as an index to measure the contribution of oxidative DNA damage to the development of antibiotic resistance (Hershberg et al., 2008). If the oxidative DNA damage contributes to DNA mutation, this ratio will increase significantly. In this study, we calculated the ratio as 0.78, which indicated that oxidative DNA damage is a dominant source of mutation for mycobacteria (Fig. 2E). A similar ratio (0.67) was also reported in mutations of *M. tuberculosis* isolated from macaques with latent infection (Ford et al., 2011). Additionally, previous studies have shown that the ratio of G:C > T:A transversion to G:C > A:T transition that confers resistance to anti-TB drugs is 0.71 (Wang et al., 2013), which is similar to our results (0.78). However, in our study, the MICs of various antibiotics did not differ between wild type mc^2^155 and mc^2^51 except that of INH, a prodrug that needs bacterial ROS activation. The results suggested that the mutation ratio is an effect rather than a cause for the acquisition of antibiotic resistance.

As ROS is correlated with *M. tuberculosis* pathogenesis, we explored whether mycobacteria obtain any benefits when evolving to adjust their resistance to H_2_O_2_. Our results showed that endogenous H_2_O_2_ resistance confers bacterial growth advantages. Newly identified genes, such as *carD*, *moaD1*, and *pp54*, involved in oxidative stress, have been shown to be important for bacterial survival inside host cells (Mestre et al., 2013). A correlation has also been shown between resistance to oxidative stress and survival in macrophages. In line with this hypothesis, our results showed that H_2_O_2_-resistant strain mc^2^51 had a statistically significant growth advantage in macrophages compared with wild-type mc^2^155 (Fig. 2D). Consistent with a previous study on the relationship of Fur to INH and KatG, the resistance of H_2_O_2_ was partially abrogated when wild type Fur was expressed in mc^2^155 (Table S3). In addition, MS data showed that mc^2^51 had increased enzyme activity of KatG (Fig. 2F). Moreover, the SNPs in Fur were also identified in INH-resistant isolates (Ramaswamy et al., 2003). This finding implies that mutant Fur could be produced in humans. Thus, these findings support our hypothesis that the evolutional adaptation of mycobacteria to H_2_O_2_, accompanied by slow growth, confers growth benefits within macrophages on the mycobacteria.

## Electronic supplementary material

Below is the link to the electronic supplementary material.

Supplementary material 1 (PDF 52 kb)

Supplementary material 2 (PDF 152 kb)
