# Supplementary material for: The gain of hydrogen peroxide resistance benefits growth fitness in mycobacteria under stress
Source: Protein Cell. 2014 Feb 22;5(3):182–5. doi: 10.1007/s13238-014-0024-5 (PMC3967060; doi:10.1007/s13238-014-0024-5)
Supplement: Supplementary file 1 — Supplementary material 1 (PDF 52 kb) [file 13238_2014_24_MOESM1_ESM.pdf]

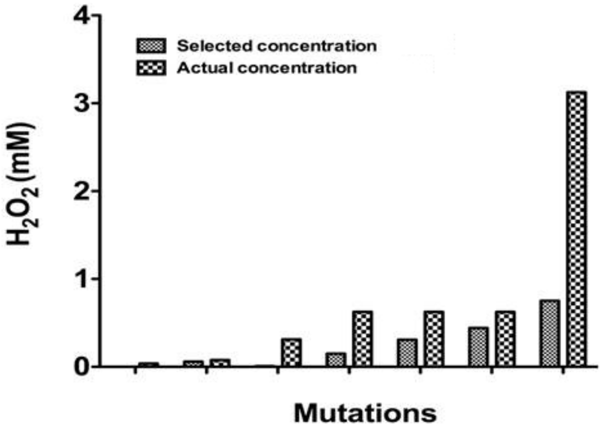

Figure S1. MIC of H_2O_2 in resistant mutants of *M. smegmatis*. The actually MICs of H_2O_2 in different mutants was compared with the corresponding selected indicated H_2O_2 concentration.
