# Supplementary material for: The gain of hydrogen peroxide resistance benefits growth fitness in mycobacteria under stress
Source: Protein Cell. 2014 Feb 22;5(3):182–5. doi: 10.1007/s13238-014-0024-5 (PMC3967060; doi:10.1007/s13238-014-0024-5)
Supplement: Supplementary file 2 — Supplementary material 2 (PDF 152 kb) [file 13238_2014_24_MOESM2_ESM.pdf]

Table S1. The MICs of anti-TB drugs of $mc^2 51$ and $mc^2 155$;

MIC	INH (mg/L)	H_2O_2 (mM)	STR (mg/L)	EMB (mg/L)	RFP (mg/L)	CIP (mg/L)	Kan (mg/L)	Amp (mg/L)
$mc^2 155$	10	0.05	0.25	0.25	5	0.125	0.625	31.25
$mc^2 51$	0.1	2.5	0.25	0.25	5	0.125	0.625	125

Table S2.Mutations identified in $mc^2 51$;

Mutation position	Gene	Protein	Reference nucleotide	Substitute nucleotide	Reference amino acid	Substitute amino acid
**185148**	**MSMEG_0161**	**formate dehydrogenase subunit alpha**	**G**	**A**	**Cys**	**Tyr**
**272276**	**MSMEG_0241**	**MmpL11**	**A**	**T**	**Tyr**	**Stop**
**934523**	**MSMEG_0850**	**hypothetical protein**	**C**	**T**	**Ala**	**Val**
1475729	MSMEG_1375	tagatose-1,6-bisphosphate aldolase, GatY	G	A	-	-
1693605	MSMEG_1606	benzoylformate decarboxylase	C	T	-	-
**3501569**	**MSMEG_3430**	**SAM-dependent methyltransferase**	**G**	**A**	**Asp**	**Asn**
3526423			C	T		
**3526518**	**MSMEG_3460**	**ferric uptake regulation protein, FurB**	**C**	**T**	**Ala**	**Val**
**3667399**	**MSMEG_3603**	**oxidoreductase, zinc-binding dehydrogenase**	**G**	**A**	**Ala**	**Val**
**3919450**	**MSMEG_3847**	**tyramine oxidase, TynA**	**G**	**T**	**Cys**	**Stop**
4212322			C	A		
4214321	MSMEG_4134	pseudogene	G	T		
4214326	MSMEG_4134	pseudogene	C	A		
4214347	MSMEG_4134	pseudogene	A	C		
5118221	MSMEG_5021	alcohol dehydrogenase	G	A	-	-
**5141995**	**MSMEG_5044**	**ATPase**	**G**	**T**	**Ser**	**Ile**
**5295214**	**MSMEG_5197**	**long-chain specific acyl-CoA dehydrogenase**	**T**	**C**	**Asp**	**Gly**
5584311			A	C		
**5953123**	**MSMEG_5893**	**hypothetical protein**	**G**	**C**	**Gln**	**Glu**
**5979886**	**MSMEG_5920**	**FMN-dependent monooxygenase**	**T**	**C**	**Asp**	**Ala**
**6915055**	**MSMEG_6866**	**peptide ABC transporter permease**	**C**	**A**	**Ala**	**Glu**
6915059	MSMEG_6866	peptide ABC transporter permease	G	T	-	-

Table S3.The effects of Fur overexpression on the INH and H_2O_2 resistance in $mc^2$51.

MIC	$mc^2$155	$mc^2$51		
	pMV361	pMV361	pMV361-L-fur	pMV361-fur
H_2O_2(mM)	<0.0244	3.125	0.3906	0.3906
INH(mg/L)	6.25	0.0195	0.3906	1.526

Table S4. Strains and Plasmids used in this study

Name	Relevant genotype or description	Reference
**Strains**		
M. smegmatis		
mc^2155	wild type	W.R. Jacobs
mc^251	An high H_2O_2 resistance strain	This work
mc^2155 (pMV261)	mc^2155 containing pMV261 vector	This work
mc^2155 (pMV361)	mc^2155 integrating pMV361 vector	This work
mc^251 (pMV261)	mc^251 containing pMV261 vector	This work
mc^251 (pMV361)	mc^251 integrating pMV361 vector	This work
mc^251 (pMV361-L-*fur*)	mc^251 integrating pMV361-L-*fur* vector	This work
mc^251 (pMV361-*fur*)	mc^251 integrating pMV361 -*fur* vector	This work
E. coli		
DH5α	F− recA1 hsdR17 thi-1 gyrA96 supE44 endA1relA1 recA1 deoR Δ(lacZYA-argF)U169 (φ80lacZΔM15)	
**Plasmids**		
pMV261	Mycobacterial extrachromosomal expression vector, Km^R	(1)
pMV361	Mycobacterial integrative expression vector, Km^R	(1)
pMV361-L-*fur*	pMV361 containing full sequence of *fur* and its 39 nt 5'-UTR, Km^R	This work
pMV361-*fur*	pMV361 containing full sequence of *fur*, Km^R	This work

(1) Stover,C.K., de la Cruz,V.F., Fuerst,T.R., Burlein,J.E., Benson,L.A., Bennett,L.T., Bansal,G.P., Young,J.F., Lee,M.H., Hatfull,G.F. et al. (1991) New use of BCG for recombinant vaccines. Nature, 351, 456–460

Table S5. Primers used in this study

**Primers**	
Name	5’-3’ sequence
Msmeg0161mvF	GCCTCAATTGCGCATATGACACTGCTCTCAGAACCCGA
Msmeg0161mvR	GCTGAAGCTTAGTGGTGATGGTGATGGTGCCTCACCAG TTCTGTCTC
Msmeg0241mvF	GCCTCAATTGCGCATATGATGCGCTTGAGCAGCACTTTG
Msmeg0241mvR	GCTGAAGCTTAGTGGTGATGGTGATGGTGCTTCGCCTCC TCCAGCATTG
Msmeg0850mvF	GCCTCAATTGCGCATATGGGCGAGGTGGTCATCGGCGGAG
Msmeg0850mvR	GCTGAAGCTTAGTGGTGATGGTGATGGTGGTGCAATCTCG GCGCCAGCCAC
Msmeg1375mvF	GCCTCAATTGCGCATATGCCGCTGGTCACCACGGCCGAG
Msmeg1375mvR	GCTGAAGCTTAGTGGTGATGGTGATGGTGGGAGGACAC CACCGTCAGAAG
Msmeg1606mvF	GCCTCAATTGCGCATATGTCCGATCAGAAGACCGTTC
Msmeg1606mvR	GCTGAAGCTTAGTGGTGATGGTGATGGTGGCCGAGC TGGGGCAGCTG
Msmeg3430mvF	GCCTCAATTGCGCATATGGTCGAGCAGAGTCTCTGGATG
Msmeg3430mvR	GCTGAAGCTTAGTGGTGATGGTGATGGTGCGCGCGACG CAGAATTGCGAC
Msmeg3460mvFL	ACGTGAATTCTAGGACTTGACTGCGTCCAATC
Msmeg3460mvF	ACGTGAATTCCGCATATGCCCTCTCGGGCGGAGTTCGAAG
Msmeg3460mvR	ACGTAAGCTTAGTGGTGATGGTGATGGTGAGGGCCGTCCA CCGAGCA
Msmeg3603mvF	GCCTCAATTGCGCATATGGTAGGAACCGAGATCAGAAG
Msmeg3603mvR	GCTGAAGCTTAGTGGTGATGGTGATGGTGTGACGGGTT TCCCATCGATAC
Msmeg3847mvF	GCCTCAATTGCGCATATGGTGTGGTTGCGTGATCGTGTG
Msmeg3847mvR	ACGTAAGCTTAGTGGTGATGGTGATGGTGGTGGTGCGC GGCCTTGGTATG
Msmeg5021mvF	GCCTCAATTGCGCATATGAAAGCTGCCGTCTATCACGGAC
Msmeg5021mvR	GCTGAAGCTTAGTGGTGATGGTGATGGTGGATGGCCGGG TCGATGAGGAC
Msmeg5044mvF	GCCTCAATTGCGCATATGTTCGTCACCGAGGACGGCGGT
Msmeg5044mvR	GCTGAAGCTTAGTGGTGATGGTGATGGTGGGTGTTCAGG GCGTCGGGTAG
Msmeg5197mvF	GCCTCAATTGCGCATATGGTGAAGCGACTCATCTACAAC
Msmeg5197mvR	GCTGAAGCTTAGTGGTGATGGTGATGGTGGACACCCAG GTCGCGGCCGATG
Msmeg5893mvF	GCCTCAATTGCGCATATGCGCTCCAAGCTCGTCGGGGCGCTG
Msmeg5893mvR	GCTGAAGCTTAGTGGTGATGGTGATGGTGGATCGGCTTGATG GCGTCGATCAG
Msmeg5920mvF	GCCTCAATTGCGCATATGAAGTTGGGTTTGCAACTC

Msmeg5920mvR	GCTGAAGCTTAGTGGTGATGGTGATGGTGCAGCAG CGCGGCGAGTTC
Msmeg6866mvF	GCCTCAATTGCGCATATGGCGATGACTGACCTGTTG
Msmeg6866mvR	GCTGAAGCTTAGTGGTGATGGTGATGGTGTCGGCC GAGCCTCACTCTC
